# New surgical techniques and surgical site infections.

**DOI:** 10.3201/eid0702.010213

**Published:** 2001

**Authors:** S M Gordon

**Affiliations:** Cleveland Clinic Foundation, Cleveland, Ohio, USA. gordons@ccf.org

## Abstract

Technologic advances in surgery include a trend toward less invasive procedures, driven by potential benefits to patients and by health-care economics. These less invasive procedures provide infection control personnel opportunities for direct involvement in outcomes measurement.


### New Surgical Techniques and Surgical Site Infections

**Steven M. Gordon**
Cleveland Clinic Foundation, Cleveland, Ohio, USA

Technologic advances in surgery include a trend toward less invasive procedures, driven by potential benefits to patients and by health-care economics. These less invasive procedures provide infection control personnel opportunities for direct involvement in outcomes measurement.

"Pray before surgery, but remember
God will not alter a faulty incision."
Arthur H. Keeney

The 21st century advancements in genetics, nanotechnology (mechanical engineering on a molecular scale), and robotics could revolutionize medical therapy and diagnostics. I will review current and future directions of minimally invasive surgery, with an emphasis on cardiac surgery, and surgical site infections after minimally invasive valve procedures.

### Minimally Invasive Surgery

Since the first endoscopic cholecystectomy was performed in France in 1988, minimally invasive surgical techniques have dramatically affected many surgical subspecialties, driven by advances in port access and video instrumentation and the desire to lessen incision pain and length of hospital stay. Advances in laparoscopic kidney and adrenal surgery now include 2-mm needle optics and instruments, which have resulted in decreased postoperative illness and superior cosmetic results (1). The challenge is to evaluate the safety and efficacy of these new techniques as they are widely introduced in the United States.

Minimally invasive cardiac surgery was predated by innovations in general surgery and is increasingly applied to cardiac procedures (30,000 worldwide in 1998). Coronary artery bypass grafting (CABG) through a median sternotomy incision with cardiopulmonary bypass support remains standard because it provides the surgeon with good exposure, a bloodless and motionless field, and myocardial protection, with graft patency rates of 90% at 10 years (2). However, cardiopulmonary bypass support may have adverse physiologic consequences, including a 6% incidence of central nervous system events (3).

There is no internationally accepted case definition for minimally invasive cardiac surgery, but two approaches to revascularization have been developed: the off-pump (beating heart) CABG, or minimally invasive direct coronary artery bypass (MIDCAB), and the endoscopic (port access technique) CABG (HeartPort, Redwood City, CA) (4).

Coronary artery anastomosis on a beating heart was first described by Kosselov in 1967 and has been modified with the MIDCAB technique to an 8-cm right or left anterior thoracotomy incision that allows direct visualization of the beating heart through small incisions. The primary candidate for this procedure is a patient with single anterior vessel disease; an estimated 1 of 3 coronary revascularization procedures (CABG or percutaneous coronary artery angioplasty) meet this criterion. The technical constraints of the MIDCAB procedure include a moving surgical field and a turgid heart on which to perform grafting. Stabilizers to control heart movement are used to facilitate anastomosis of the target grafts during suturing.

The port-access operation involves a mini-thoracotomy (8 cm) on an arrested heart by using percutaneously inserted endovascular occluder balloons in the ascending aorta. Unlike port-access surgery in noncardiac surgical subspecialties, almost all cardiac operations on adult patients are reconstructions that are technically more demanding when performed through an endoscope. In addition, the laparoscopic approach with an insufflated peritoneum provides better exposure than open techniques (5).

Surgical site infections after minimally invasive cardiac surgery pose a challenge to the clinician. Physical findings of sternal instability and sternal click of the median sternotomy cannot be applied to many incisions used in minimally invasive cardiac surgery (Figure 1). The initial experience of

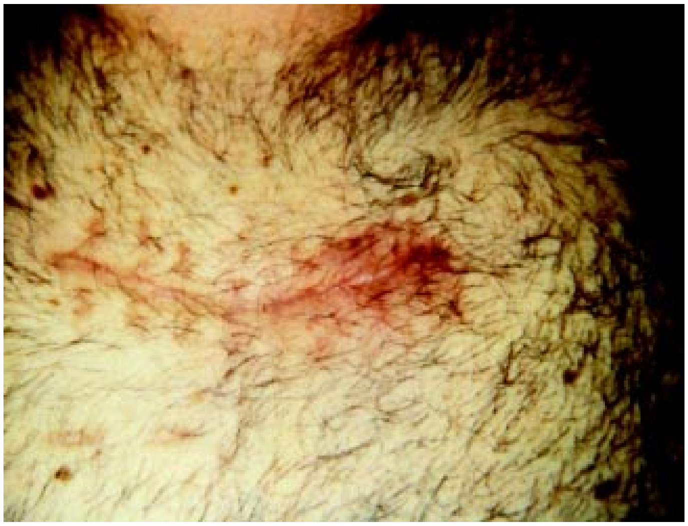

Figure 1. Surgical site infection following minimally invasive valve surgery.

Address for correspondence: Steven M. Gordon, Department of Infectious Diseases, Cleveland Clinic Foundation, 9500 Euclid Avenue, Mailstop S-32, Cleveland, Ohio, 44195, USA; fax: 216-445-9446; e-mail: gordons@ccf.org

1,400 minimally invasive cardiac surgery procedures at the Cleveland Clinic showed no significant difference in the incidence of deep or superficial wound infections (Table).

Table. Rates[a] of surgical site infections in patients undergoing minimally invasive compared with traditional open heart surgery, Cleveland Clinic Foundation, 1996–98

| | Traditional (n=9,633) | Minimally invasive (n=1,400) | p value >0.05 |
|---|---|---|---|
| Overall rate[a] | 3.3 | 2.9 | Not significant |
| Deep infection | 1.7 | 1.9 | Not significant |

[a]per 100 procedures

An important quality indicator for minimally invasive surgical procedures is the conversion rate to open procedures. A surgeon's decision to convert from a minimally invasive procedure to an open procedure may be determined by poorly defined anatomy or surgical complications. Conversion is not necessarily a failure but may be used as a quality indicator, and conversion rates for minimally invasive cardiac surgery procedures have declined substantially with increasing experience at our institution (Figure 2). The introduction of

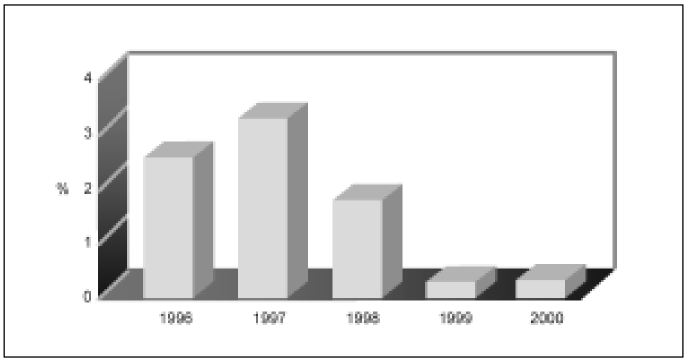

Figure 2. Conversion rates to open procedures among patients undergoing minimally invasive heart surgery, Cleveland Clinic Foundation.

any new surgical technique involves a learning curve, and increased experience may be translated into reduced illness and death. Examples of the relationship between surgeon-specific volume and death associated with CABG procedures have been published (6-8).

The association of outcome with case volume may not depend on a single person but on the collective abilities of the clinical team (9). High volumes may also reflect selection bias by patient referrals to institutions and surgeons with good outcomes. Health-care consumers are increasingly interested in outcome measurements, and one consumer advocate group (the Center for Medical Consumers) has compiled 1998 data from the New York State Department of Health for 21 surgical procedures, stratified by volume, hospital, and individual practitioner (available at URL www.medicalconsumers.org).

### Solid Organ Transplantation

The greatest challenge facing solid organ transplantation in the United States is a shortage of donors, with approximately three persons awaiting transplantation for every organ donated. Organs from pigs may alleviate the shortage, but the challenge of xenotransplantation is in replacing xenogenic epitopes (antigens) recognized as foreign by the immune system. An additional concern is trans-species transmission of endogenous retroviruses from donor animals, such as porcine endogenous retrovirus (PoERV). Two cases of successful extracorporeal hepatic support with transgenic pig livers have been reported with no evidence of human PoERV infection at 5 and 185 months of follow-up (10).

Another alternative to cardiac allotransplantation is the implantable ventricular assist device (11). The two types approved by the U.S. Food and Drug Administration (HeartMate left ventricular assist device, ThermoCardiosystems, Woburn, MA, and the Novacor left ventricular assist device, World Health, Inc., Oakland, CA) are both electrical pulsatile devices, implanted through a median sternotomy with an inflow cannula in the apex of the left ventricle and an outflow tube anastomosed to the ascending aorta. A single drive line containing the electrical cable and the atmospheric air vent leads transcutaneously from the implanted pump to an external power pack (Figure 3).

Recipients of implantable left ventricular assist devices are vulnerable to device-related infections because the extracorpeal drive line (13.5 mm to 15 mm in diameter) breaches normal cutaneous defenses against infection, providing a portal of entry for pathogens (12). The incidence of infection increases with duration of ventricular assist device support (a mean of 120 days for patients awaiting heart transplantation at the Cleveland Clinic in 1999). As recipients are often malnourished or debilitated, it is not surprising that 32% of patients had a device-associated infection and 55% had a hospital-associated bloodstream infection during support (13). Patients with ventricular assist devices commonly receive antibiotic therapy, both for prophylaxis or treatment of infections and on an empiric

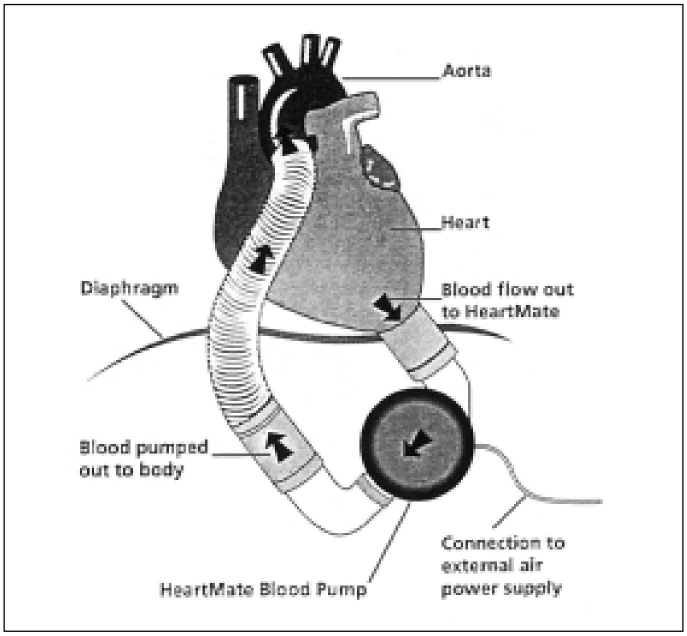

Figure 3. Implantable left ventricular assist device.

basis. The use of antibiotics may lead to development of infections with fungi and drug-resistant pathogens. Despite these implications, infections associated with ventricular assist devices do not preclude successful transplantation. Strategies for prevention of infection in recipients will focus on the drive line exit site until technical advances can achieve a totally implantable device.

### Future Directions: From Blood and Guts to Bits and Bytes

Technologic advances are continually being brought into the operating room with increasing use of robotics and teleoperating systems and virtual environment, which is the fusion of robotics and three-dimensional imaging technology. One issue with laparoscopic surgery is control of the camera (laparoscopic lens) while the surgeon operates. There may be problems with second guessing where the surgeon wants the camera lens directed; movement of the camera lens, leading to iatrogenic complications; and the expense of additional personnel. Voice activation of a surgical robotic assistant has permitted single-surgeon thorascopic surgery (14). The surgeon registers voice commands into a voice card, and the thorascope is connected with a robotic arm. In a study of human-assisted versus robotic-assisted surgeries, all procedures were successfully completed with no difference in operating times and no technical mishaps related to the robot.

Teleoperating systems and telesurgery allow the operator to perform surgery from a remote site. A three-dimensional camera is outfitted with tactile, auditory, and proprioceptive feedback. This technology may provide a means to treat patients in hazardous or distant environments where evacuation is not feasible. NASA is planning to send astronauts on a 3-year mission to Mars by 2020 and believes an acute medical crisis is likely during such a voyage. Biomedical space researchers are reviewing the creation of a digitized virtual astronaut, a computerized representation of the entire physiology, updated in real time by input from a comprehensive bank of sensors (Groopman J. Medicine on Mars. New Yorker, February 14, 2000). Any necessary surgery would be performed by the flight surgeon, coached by the virtual mentor and aided by robotics.

In summary, the operating room remains a dynamic environment undergoing rapid change and innovation. The challenge for infection control practitioners is to adopt a facilitative (not passive or resistant) involvement in measurement and data-tracking instruments (e.g., registries, conversion rates, surgical site infection rates) and embrace opportunities for comparison.

Dr. Gordon is hospital epidemiologist and infectious disease staff physician at the Cleveland Clinic Foundation and former Epidemic Intelligence Service Officer (class of 1987) in the Hospital Infections Program, CDC.

### References

1. Gil IS, Soble JJ, Sung GT, Winfield HN, Bravo EL, Novick AC. Needlescopic adrenalectomy: the initial series—comparison with conventional laparoscopic adrenalectomy. Urology 1998;52:180-6.
2. Loop FD, Lytle BW, Cosgrove DM, Stewart RW, Goormastic M, Williams GW, et al. Influence of the internal-mammary-artery graft on 10-year survival and other cardiac events. N Engl J Med 1986;314:1-6.
3. Roach GW, Kanchuger M, Mangano CM, Newman M, Nussmeier N, Wolman R, et al. Adverse cerebral outcomes after coronary artery bypass surgery. N Engl J Med 1996;335:1857-63.
4. Sabik J. The keyhole or the manhole? What internists need to know about minimally invasive CABG. Cleveland Clinic J of Med 1998;65:454-6.
5. Lytle BW. Minimally invasive cardiac surgery. J Thorc Cardiovasc Surg 1996;111:554-5.
6. Showstack JA, Rosenfeld KE, Gaarnick DW, Luft HS, Schaffarzick RW, Fowles J. Association of volume with outcome of coronary artery bypass graft surgery. JAMA 1987;257:785-9.
7. Hannan EL, O'Donnell JF, Kilburn H, Bernard HR, Yazici A. Investigation of the relationship between volume and mortality for surgical procedures performed in New York State hospitals. JAMA 1989;262;503-10.
8. Hannan EL, Kilburn H, Racz M, Shields E, Chassin MR. Improving the outcomes of coronary artery bypass surgery in New York State. JAMA 1994;271:761-6.
9. Laffel GL, Barnett AI, Finklestein S, Kaye MP. Relationship between experience and outcome in heart transplantation. N Engl J Med 1992;327:1220-5.
10. Levy MF, Crippin J, Sutton S, Netto G, McCormack J, Curiel T, et al. Liver allotransplantation after extracorporeal hepatic support with transgenic (hCD55/hCD59) porcine livers. Transplantation 2000;69:272-80.
11. Goldstein DJ, Oz HC, Rose EA. Implantable left ventricular assist devices. N Engl J Med 1998;339:1522-33.
12. McCarthy PM, Schmitt SK, Vargo RL, Gordon SM, Keys TF, Hobbs RE. Implantable LVAD infections: implications for permanent use of the device. Ann Thorac Surg 1996;61:3590-5.
13. Schmitt SK, Serkey J, McCarthy PM, Gordon SM. Infections in patients on LVAD: The Cleveland Clinic experience [Abstract #9]. Annual Meeting of Society for Healthcare Epidemiology of America; April 4-7, 1995; San Diego, California.
14. Okada S, Tanaba Y, Yamauchi H, Sato S. Single-surgeon thorascopic surgery with a voice-controlled robot. Lancet 1998;351:1249.
